# Scientific

**Published:** 1889-11

**Authors:** 


### SCIENTIFIC ITEMS.

Edison says, "Electricity is the coming motive power." He is working at the problem of converting heat directly into electricity without the intervention of steam, which is expansive. He believes it will be done. The Weems system of transportation promises that through electrical machinery the New York papers will be read in Pittsburg at the breakfast table on the morning of their publication. A company has been organized in Baltimore to put this system into active operation.

Experiments again made in London with carbo-dynamite, one of the latest explosives, would seem to show that it possesses some important advantages over ordinary dynamite, among others that of considerably greater power and the generation of much less noxious vapor when exploded in confined places. It is composed of nitro-glycerine absorbed by ten parts of a variety of carbon, and is claimed to be entirely unaffected by water.

At Pocasset, Mass., a large business is growing up in the manufacture of metal ornaments by electric deposit. Moulds of a wax composition are black-leaded and hung in the metallic bath, connected with a powerful dynamo, and, after thirty-six hours, come out with copper or silver on them twice the thickness of sheet zinc, and only needing touches of coloring matter to finish them for the market.

Mr. W. A. Ashe, of Quebec, reports that the Esquimaux living near Hudson Strait have a mean height for the men of 5 feet 3.9 inches, and for the women about 5 feet. Their body temperature averaged 100.2 degrees for winter and 98.4 degrees for summer, that of the observing party being 98.1 degrees and 97.7 degrees respectively.—*Ex.*

The average monthly temperature of San Francisco for the last fifteen years has been 55¼ degrees. The highest for any month was 59 degrees, and the lowest 50 degrees.—*Ib.*

**Combined Phonograph and Photograph.**—At a recent meeting of the French Academy M. Lippmann presented a note by M. G. Gueroult, in which it is suggested that by the combined use of a phonograph and an apparatus for instantaneous photography and reproduction of the pictures obtained, it would be possible to reproduce at any future time not only the future speech of a person, but also bring before the audience a vivid picture of the person's gestures and facial expression.

The procedure would be somewhat as follows: A person speaking or singing into the phonograph would be photographed by automatic apparatus geared with the barrel of the phonograph. The pictures would be instantaneous, and taken at the rate of, say, ten pictures per second. They would then be developed and arranged in a special lantern for reproduction on a screen isochronously with the phonograph, when the latter is producing the speech. An audience might thus be enabled not only to hear the utterances of, say, a famous actor, but also see himself and his actions represented on a screen. About a year and a half ago M. A. Bandsept, of Brussels, experimented with a similar apparatus.—*Scientific American.*

**Extent and Population.**—The United Sates of America, which separated from England in 1776, and elected their first president in 1789, now consist of 42 States, 6 Territories, and 1 Federal District. The total area of the union, including Alaska, is about 3,605,000 square miles. As for the population, that during the century has made a truly fabulous progress.

While Great Britain's population has, in fifty years, increased by 10 millions, France's by 5 mllions, Germany's by 16 millions, the population of the United States has increased 37 millions. It has been calculated that, since 1790, the population of North America has been doubling about every 26 years. At present the population of the American Union must certainly exceed 62 million inhabitants. Now, in 1790 the population did not reach 4 millions. In one century, then, the population has varied in the proportion of 1 to 15.5.

If this ascending advance continues, and there is every evidence that it will, the United States in 50 years will count more than 200 million inhabitants, and in 70 years will be as populous as Europe.

**Cooking by Electricity.**—The Hotel Bernina, at Samaden, has for some time been lighted with electricity, power being supplied by a waterfall. As during the day the power is not required for lighting, and is therefore running to waste, the proprietor of the hotel has hit upon the idea of utilizing the current for cooking when it is not required for lighting, and an experimental cooking apparatus has been constructed. This contains German silver resistance coils, which are brought to a red heat by the current, and it has been found possible to perform all the ordinary cooking operations in a range fitted with a series of such coils.—*Ib.*

**85 Miles in 82 Minutes.**—The New York delegation of the Ancient Order of Foresters lately arrived at Minneapolis to attend the National Convention there. They traveled in a special train, and on one part of the journey made the remarkable speed of 85 miles in 82 minutes or at the rate of over 62 miles per hour.—*Ib.*

**Improvement on the Phonograph.**—In the present phonograph, a stylus for impressing the wax is attached to the center of the vibrating diaphragm. The new improvement of G. Bettini is to extend little rods from the stylus to several parts of the diaphragm. In this way greater exactness of tone and speech is obtained, so the inventor claims, and much superior results.—*Ib.*

